# ACE2‐enriched extracellular vesicles enhance infectivity of live SARS‐CoV‐2 virus

**DOI:** 10.1002/jev2.12231

**Published:** 2022-05-18

**Authors:** Sze Keong Tey, Hoiyan Lam, Samuel Wan Ki Wong, Hanjun Zhao, Kelvin Kai‐Wang To, Judy Wai Ping Yam

**Affiliations:** ^1^ Department of Pathology School of Clinical Medicine Li Ka Shing Faculty of Medicine The University of Hong Kong, Hong Kong Special Administrative Region Pokfulam People's Republic of China; ^2^ School of Biological Sciences College of Science Nanyang Technological University Singapore Singapore; ^3^ State Key Laboratory for Emerging Infectious Diseases, Carol Yu Centre for Infection, Department of Microbiology, School of Clinical Medicine, Li Ka Shing Faculty of Medicine The University of Hong Kong, Hong Kong Special Administrative Region Pokfulam People's Republic of China

## 1 INTRODUCTION

Extracellular vesicles (EVs) are nanoscale vesicles naturally released from cells that are delimited by a lipid bilayer and cannot replicate (Van Niel et al., 2018). Growing evidence has revealed the importance of EVs in intercellular communication by transferring bioactive molecules, including nucleic acids, proteins and lipids, from donor cells to recipient cells (Mathieu et al., 2019). Since then, EVs have been shown to be implicated in both physiological and pathological processes (Shah et al., 2018; Yáñez‐Mó et al., 2015). Recently, a new role is emerging for EVs as the playmaker of viral transmission. For example, non‐enveloped hepatitis A virus (HAV) has been found to exploit EV biogenesis pathways, particularly ESCRT proteins, to exit cells in an enveloped state (Feng et al., 2013). Another non‐enveloped hepatitis E virus (HEV) has also been reported to exist within EVs and to be released in an enveloped state (Nagashima et al., 2014). Moreover, enterovirus packaged in EVs can infect host cells at a greater efficiency than free viral particles (Chen et al., 2015). Other types of viruses that are utilizing EVs as viral transmission routes have been comprehensively reviewed elsewhere (Alenquer & Amorim, 2015; Altan‐Bonnet, 2016; Urbanelli et al., 2019).

Since December 2019, a novel coronavirus infectious disease caused by severe acute respiratory syndrome coronavirus 2 (SARS‐CoV‐2) has become a pandemic and named as coronavirus disease 2019 (COVID‐19) by World Health Organization (WHO) (Zhou et al., 2020; Zhu et al., 2020). The main entry route of SARS‐CoV‐2 into host cells is mediated by the interaction of viral surface spike (S) protein with the surface receptor angiotensin‐converting enzyme 2 (ACE2) (Benton et al., 2020; Yan et al., 2020). ACE2 is widely expressed at the surface of human airway and intestinal epithelial cells, thus potentiating these cells to be highly susceptible to SARS‐CoV‐2 infection (Ziegler et al., 2020). Mechanistically, the full length spike precursor is cleaved into S1 and S2 by furin during release from infected cells (Walls et al., 2020). Upon binding to ACE2, the S2 subunit of S protein is cleaved by transmembrane protease serine 2 or 4 (TMPRSS2 or TMPRSS4) at the S2’ site and allows viral entry into host cells (Hoffmann et al., 2020; Zang et al., 2020). As ACE2 also present in soluble form after proteolytic cleavage (sACE2), attempts have been made to utilize high dosage of human recombinant sACE2 to inhibits SARS‐CoV‐2 infection using in vitro model (Monteil et al., 2020). Intriguingly, a recent study has showed that sACE2 at physiological range indeed mediates cell entry of SARS‐CoV‐2, suggesting the dual role of sACE2 in SARS‐CoV‐2 infection (Yeung et al., 2021). Here, we explore the possibility whether surface ACE2 carried by EVs can facilitate the SARS‐CoV‐2 infection into cells.

## 2 MATERIAL AND METHODS

### 2.1 Cell lines

293T (Cat# CRL‐3216) and Vero E6 (Cat# CRL‐1586) cell lines were purchased from American Type Culture Collection. 293T cell overexpressing ACE2 was prepared by transfecting pcDNA3.1‐ACE2 plasmid as described previously (Li et al., 2003). In brief, 5 × 10^5^ 293T cells were transfected with 1 μg ACE2‐expressing plasmid (ACE2‐OE) with Lipofectamine 2000 (Invitrogen) and 293T transfected with pcDNA3.1 empty vector was used as control (CTL). 293T and Vero E6 cells were cultured in Dulbecco's Modified Eagle Medium (DMEM, Gibco) supplemented with 10% foetal bovine serum (FBS, Gibco), 100 IU/ml penicillin (Gibco) and 100 μg/ml streptomycin (Gibco) at 37°C and 5% CO_2_. All cell lines were tested routinely before use to avoid mycoplasma contamination.

### 2.2 Isolation of EVs from conditioned medium of cell culture

Extracellular vesicles were collected from conditioned medium as described previously (Mao et al., 2020). In brief, cells were cultured in 10% EV‐depleted FBS (Gibco) supplemented media for 72 h prior to EV collection. To prepare EV‐depleted FBS, FBS was centrifuged overnight at 100,000 × g at 4°C (Beckman Coulter, Optima XPN‐100, Type 45 Ti, k‐factor: 313). Conditioned medium was collected from cells cultured in EV‐depleted FBS supplemented media and EVs were purified by differential centrifugation. Medium was centrifuged at 2,000 × g for 15 min to remove cell debris and dead cells (Thermo Fisher Scientific, Heraeus Multifuge X3FR, TX‐750). Supernatant was then centrifuged at 20,000 × g for 30 min at 4°C to remove microvesicles (Beckman Coulter, Optima XPN‐100, Type 45 Ti, k‐factor: 1563). The supernatant was filtered by 0.2 μm filter with PES membrane (ThermoFisher Scientific, Cat# 595–4520) and EVs were collected by further centrifuging supernatant at 100,000 × g for 70 min at 4°C (Beckman Coulter, Optima XPN‐100, Type 45 Ti, k‐factor: 313). EV pellet was washed with phosphate‐buffered saline (PBS) and collected by ultracentrifugation at 100,000 × g for 70 min at 4°C (Beckman Coulter, Optima XPN‐100, Type 45 Ti, k‐factor: 313). All relevant data of our experiments was submitted to the EV‐TRACK knowledgebase (EV‐TRACK ID: EV210342) (Van Deun et al., 2017).

### 2.3 Extraction of EV proteins, conditioned medium preparation and western blotting

Protein lysates of EVs and cells were obtained by lysing EV with RIPA lysis buffer (Thermo Scientific, Cat#89901), supplemented with 10% cOmplete protease inhibitor cocktail (Roche Applied Science, Cat# 05892970001) and 10% PhosStop phosphatase inhibitor cocktail (Roche Applied Science, Cat# 04906837001). The protein concentration of EV lysate was determined by Bradford assay (Bio‐Rad Corporation, Cat# 5000006) using bovine serum albumin (BSA) as standard. Conditioned medium of cells was collected after 72 h incubation and centrifuged at 2,000 × g for 15 min to remove cell debris and dead cells. The medium was concentrated by using Amicon® Ultra‐15 Centrifugal Filter Unit (MWCO: 10 kDa, Millipore, Cat# UFC901024). Fifteen ml of medium was centrifuged at 3000 × g for 30 min to reach final volume of about 500 μl (30× concentration). The protein concentration of remaining concentrated samples was measured by Bradford assay. For western blotting, a total of 30 μg of protein per lane was fractionated by sodium dodecyl sulfate–polyacrylamide gel electrophoresis (SDS‐PAGE) using 7.5% or 10% gel made by TGX FastCast Acrylamide Kit (Bio‐Rad Corporation, Cat# 1610173). After electrophoresis, proteins were transferred to PVDF membranes (Cytiva, Cat# GE10600023) using Trans‐Blot Turbo System (Bio‐Rad Corporation) and subjected to western blot analysis. Chemiluminescent signals were detected by ECL Western Blotting Detection Reagents (Cytiva, Cat# GERPN2209).

### 2.4 Validation of isolated EVs

The identity of EVs was analysed for their expressions of positive and negative markers of small EVs (Jeppesen et al., 2019; Willms et al., 2016). Positive marker, TSG101 and negative markers, GM130 and α‐tubulin were analysed. Western blotting was performed using anti‐ACE2 (R&D Systems, Cat# AF933, 1 μg/ml), anti‐TSG101 (BD Biosciences, Cat# 612696, 1:1000), anti‐GM130 (Abcam, Cat# ab52649, 1:1000), αanti‐tubulin (Cell Signalling Technology, Cat# 2144, 1:1000), anti‐TMPRSS2 (Santa Cruz, Cat# sc‐515727, 1:250), anti‐TMPRSS4 (Santa Cruz, Cat# sc‐376415, 1:250). HRP‐conjugated secondary antibodies against mouse (#62‐6520, 1:5000), rabbit (#65‐6120, 1:5000) and goat (#31402, 1:5000) were purchased from Invitrogen. The size range of EVs was determined by ZetaView® TWIN NTA PMX‐220 (Particles Metrix GmbH). The device was calibrated by using standard beads (100 nm) provided by the manufacturer. To prepare EV samples for injection, 10 μl of EVs suspension was diluted in PBS (dilution factor: 1:2500‐10000). For each sample, 1 ml of diluted suspension was injected with temperature set at 25°C and pH 7.0. Automated measurements taken at 11 distinct positions with standard instrument setting (sensitivity:75, shutter: 100, min. brightness: 30; min. area: 10; max area: 1000; fps: 30) in the sample cell and the peak analysis was performed by ZetaView software (version 8.05.11).

### 2.5 Immunogold labelling of EVs

EVs were fixed with 2% PFA and incubated on formvar carbon‐coated nickel grids for 20 min at room temperature. The grids were washed three times with PBS followed by blocking with 1% BSA/PBS for 10 min. The grids were then incubated with 1:25 diluted anti‐CD63 (Abcam, Cat# ab134045) and anti‐ACE2 (R&D Systems, Cat# AF933) antibodies in 1% BSA/PBS for 30 min. After washing three times with 1% BSA/PBS for 3 min each, the grids were incubated with 10 μl of secondary antibodies conjugated with 6 nm (Abcam, Cat# ab41498) or 15 nm gold particles (Abcam, Cat# ab27247) (1:1 ratio in 1% BSA/PBS) for 20 min. Finally, the grids were washed with PBS before counterstained with uranyl‐acetate (UA) and methyl cellulose‐UA and visualized under Tecnai T12 Transmission Electron Microscope (FEI Company).

### 2.6 Fluorescence labelling of EVs for uptake assay

The isolated EVs were fluorescently labelled using PKH67 Green Fluorescent Cell Linker Kit (Sigma Aldrich, Cat# PKH67GL) according to the manufacturer's manual as described previously (Mao et al., 2020). In brief, 5 × 10^10^ ACE2‐OE‐EVs were mixed in Diluent C in final volume of 1 ml. In separate tube, 4 μl of PKH67 dye solution was added into 1 ml of Diluent C (4 × 10^–6^ M, 2× dye solution) and mix well. The EVs suspension were rapidly mixed with dye solution and incubate for 5 min at room temperature with periodic mixing. The staining was stopped by adding 8 ml of EV‐depleted complete medium and centrifuged at 100,000 × g for 70 min at 4°C (Beckman Coulter, Optima XPN‐100, Type 45 Ti, k‐factor: 313). Labelled EVs pellet was washed with PBS and collected by ultracentrifugation. PBS used as control for EV uptake assay were subjected to same staining procedure. For EVs uptake assay, 5 × 10^4^ Vero E6 cells were seeded on cover slip and allowed to adhere for 24 h. Cells were treated with DMSO (Sigma‐Aldrich, Cat# D2650), EIPA (50 μM) (Sigma‐Aldrich, Cat# A3085), Cytochalasin D (1 μM) (Sigma‐Aldrich, Cat# C2618), Filipin (10 μg/ml) (Sigma‐Aldrich, Cat# F4767), and BafA1 (10 nM) (Sigma‐Aldrich, Cat# B1793) for 30 min before incubating with 5 × 10^9^ PKH67‐labeled EVs for 2 h. After incubation, EV‐treated cells were fixed with 4% formaldehyde in PBS and stained with DAPI (Invitrogen, Cat# D1306) before examination under laser scanning confocal microscopy (Carl Zeiss LSM900). The AF488 mean intensity was analysed using ZEN Software Version 3.1.

### 2.7 Multicycle virus growth assay with or without EV

The virus was produced as described previously (Lu et al., 2021). SARS‐CoV‐2 strain HKU001a was cultured using Vero E6 cells in a biosafety level 3 facility. Briefly, 1 × 10^7^ cells were seeded with 9 ml of DMEM in T75 cell culture flask and incubated at 37°C in a carbon dioxide incubator for 1–2 days until confluence for inoculation. 0.02 ml of virus culture stock was inoculated in the cell culture flask with 9 ml of DMEM and was incubated at 37°C for 3 days. The amount of virus was quantified using plaque assay.

For the multicycle study, SARS‐CoV‐2 (1 × 10^3^ PFU/ml) was premixed in ratio 1:1 with or without EVs (EVs original concentration: 1 × 10^11^ particles/ml, diluted to 1:2, 1:4, 1:8 and 1:32) (100 μl of virus + 100 μl of diluted EVs) for 1 h in room temperature, and was then added to Vero E6 cells for incubation at 37°C. At 1 h post infection, the infectious media were removed and replaced with fresh media containing the corresponding diluted EVs. Cell supernatant was collected for reverse transcription quantitative polymerase chain reaction (RT‐qPCR) at 1 h and 24 h post infection.

For experiments involving Cytochalasin D, EIPA, Filipin and BafA1, cells were pretreated with the corresponding drugs with working concentrations (1 μM and 2 μM for Cytochalasin D and 10 nM for BafA1) for 30 min at 37°C. Mixtures of virus and EVs (1:8 dilution) were incubated for 1 h at room temperature and then added to VeroE6 cells for incubation at 37°C. At 1 h post infection, the infectious media was removed and replaced with fresh media containing EVs and the corresponding drugs. Cell supernatant was collected for RT‐qPCR at 24 h post infection.

### 2.8 RT‐qPCR

RT‐qPCR targeting the SARS‐CoV‐2 RNA‐dependent‐RNA‐polymerase (RdRp)‐helicase gene region was performed as described previously with modifications (To et al., 2020; Zhao et al., 2020). Briefly, RNA was extracted from culture supernatant using Viral RNA Mini Kit (Qiagen, Cat# 52906). RT‐qPCR targeting the SARS‐CoV‐2 RdRp‐helicase gene region was performed using QuantiNova Probe RT‐PCR Kit (Qiagen, Cat# 208352). The reagent mixture (20 μl) contained 10 μl of 2 × QuantiNova Probe RT‐PCR Master Mix, 0.2 μl of QN Probe RT‐Mix, 1.6 μl of each 10 μM forward and reverse primer, 0.4 μl of 10 μM probe, 1.2 μl of RNase‐free water and 5 μl of RNA samples as the template. The thermal cycling condition was 45°C for 10 min (reverse transcription), 95°C for 5 min (PCR initial activation), followed by 45 cycles of 95°C for 5 s and 55°C for 30 s. All reactions were performed using the LightCycler^®^ 96 System (Roche Life Science).

### 2.9 Quantification and statistical analysis

The data of all assays was calculated as mean ± standard error mean (SEM). Student's *t*‐test and multiple *t*‐test performed by GraphPad Prism 8 were used for the statistical analysis. *P*‐value of less than 0.05 was considered as statistically significant.

## 3 RESULTS

### 3.1 EVs containing ACE2 enhance infection efficiency of SARS‐CoV‐2 live virus

**FIGURE 1:**
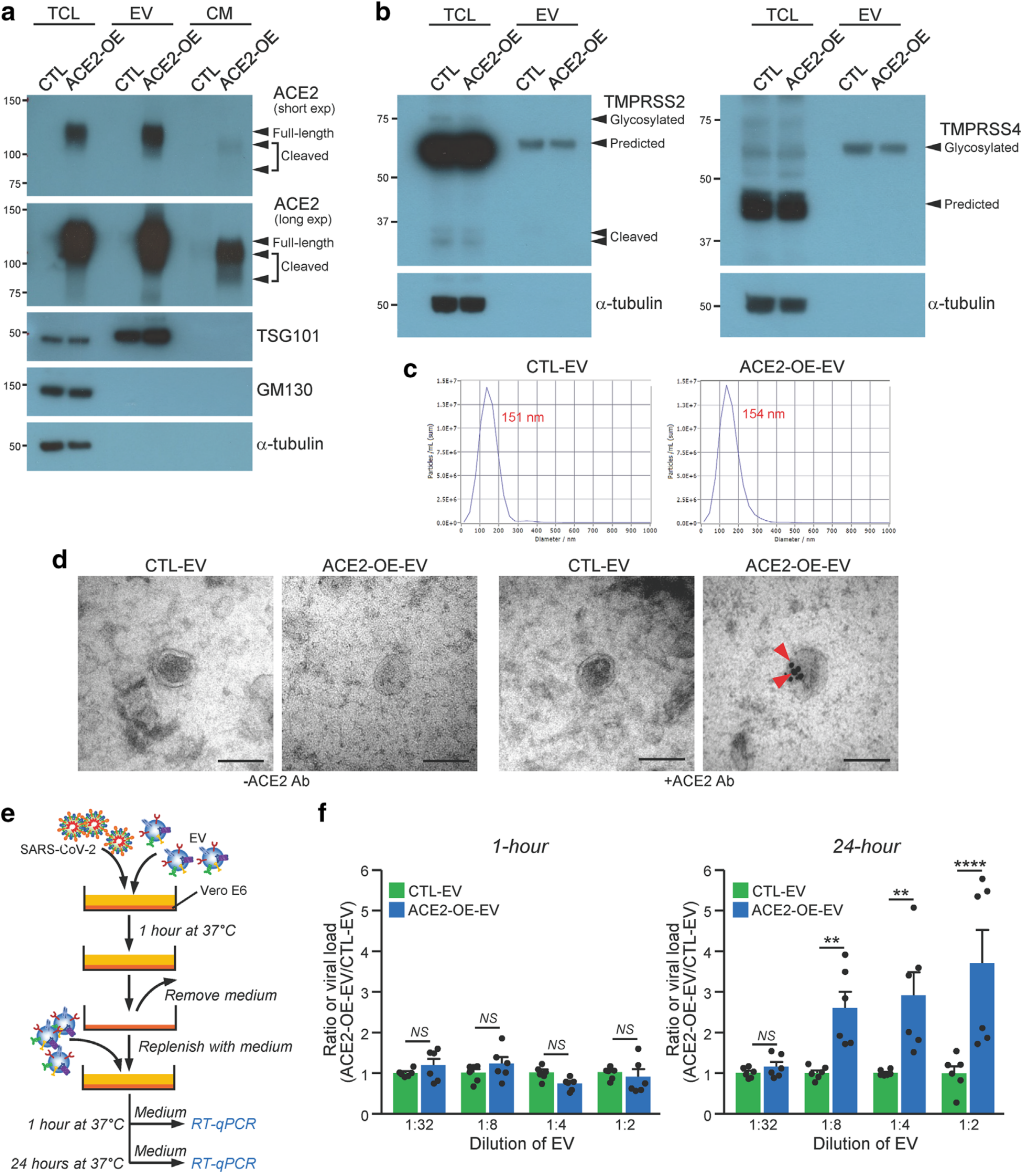
ACE2‐enriched EVs increase infectivity of live SARS‐CoV‐2 on Vero E6 cells. (a) Western blot analysis of ACE2 in the total cell lysate (TCL), extracellular vesicle (EV) and conditioned medium (CM) of control (CTL) and stable ACE2 overexpressing (ACE2‐OE) cells. Positive (TSG101) and negative (GM130 and α‐tubulin) markers of small EVs were examined. Arrowheads indicate the expressions of different forms of ACE2. (b) Expressions of TMPRSS2 (*left*) and TMPRSS4 (*right*) in control and ACE2 overexpressing cells. Arrowheads indicate the expressions of different forms of TMPRSS2 and TMPRSS4. (c) Size range of EVs was measured by nanoparticle tracking analyser. Automated measurements taken at 11 distinct positions with standard instrument setting (sensitivity:75, shutter: 100, min. brightness: 30; min. area: 10; max area: 1000; fps: 30) in the sample cell and the peak analysis was performed by ZetaView software. The mode size is displayed by font in red. (d) EVs were purified from medium of control and ACE2 overexpressing cells. EVs were subjected to immunogold labelling with or without anti‐ACE2 antibody with secondary antibodies conjugated to 15‐nm gold particles. Representative electron micrographs of EVs. Scale bar: 100 nm. (e) Schematic diagram illustrating the multicycle growth assay. (f) Vero E6 cells were infected with SARS‐CoV‐2 premixed with the indicated diluted EVs. The infectious media were collected to determine viral load by RT‐qPCR. The data represent the mean of 2 independent experiments with 3 biological samples each. Error bars represent mean ± SEM. Asterisks indicate statistical significance compared with PBS control group. **P < 0.01, ****P < 0.0001. *NS*, Not significant

To obtain EVs enriched with ACE2, 293T cells overexpressing ACE2 (ACE2‐OE) were cultured in medium containing EV‐depleted FBS and EVs were collected by ultracentrifugation (Mao et al., 2020). 293T cells transduced with empty vector were used as a control (CTL). Expression of ACE2 in total cell lysate was confirmed by immunoblotting and the enrichment of ACE2 within EVs coincident with the presence of small EV positive marker, TSG101 and absence of small EV negative markers, GM130 and α‐tubulin, were observed (Figure 1a). It is worth noting that full‐length transmembrane ACE2 was detected in cells and EVs while additional two cleaved forms of ACE2 were found in the conditioned medium. The expressions of TMPRSS2 and TMPRSS4 in control and ACE2 overexpressing cells were also analysed. Glycosylated, predicted or cleaved forms of TMPRSS2 and TMPRSS4 were detected in total cell lysates while only the predicted size of TMPRSS2 and glycosylated TMPRSS4 were present in EVs (Figure 1b). EVs isolated from both cells were further examined under nanoparticle tracking analyser. These cells released comparable number of particles and posed similar mode size (Figure 1c). The morphology of EVs was revealed by electron microscopy (Figure 1d). The presence of ACE2 on the surface of EVs derived from ACE2‐OE cells was detected by immunogold labelling using anti‐ACE2 antibody (Figure 1d). The capacity of ACE2‐enriched EVs to enhance infection was analysed by multicycle virus growth assay as illustrated (Figure 1e). The SARS‐CoV‐2 live virus was prepared as described previously (Lu et al., 2021; Zhao et al., 2020). In the multicycle growth assay, infectious virions were produced about 6 h after infection. These virus progenies could then infect other cells. The EVs that was replenished after infection would facilitate the second and subsequent rounds of infection. The cell culture supernatant after 1 h and 24 h infection was collected for RT‐qPCR to determine the infectivity. RT‐qPCR targeting the SARS‐CoV‐2 RNA‐dependent‐RNA‐polymerase (RdRp)‐helicase gene region was performed. In order to make fairer comparison, we calculated the ratio of viral load of ACE2‐OE‐EV group over CTL‐EV group, with different concentration of EVs being used. The infectivity of the Vero E6 cells by SARS‐CoV‐2 live virus mixed with ACE2‐OE‐EV increased in a concentration dependent manner after 24 h of infection (Figure 1f and Figure S1>). Though the amount of EVs was increased, the infection remained unaffected by CTL‐EVs. There was no significant difference in the infectivity of the Vero E6 cells by virus, with or without EVs, at 1 h post infection.

### 3.2 Inhibition of EVs uptake diminishes the infectious efficiency of SARS‐CoV‐2 live virus

**FIGURE 2:**
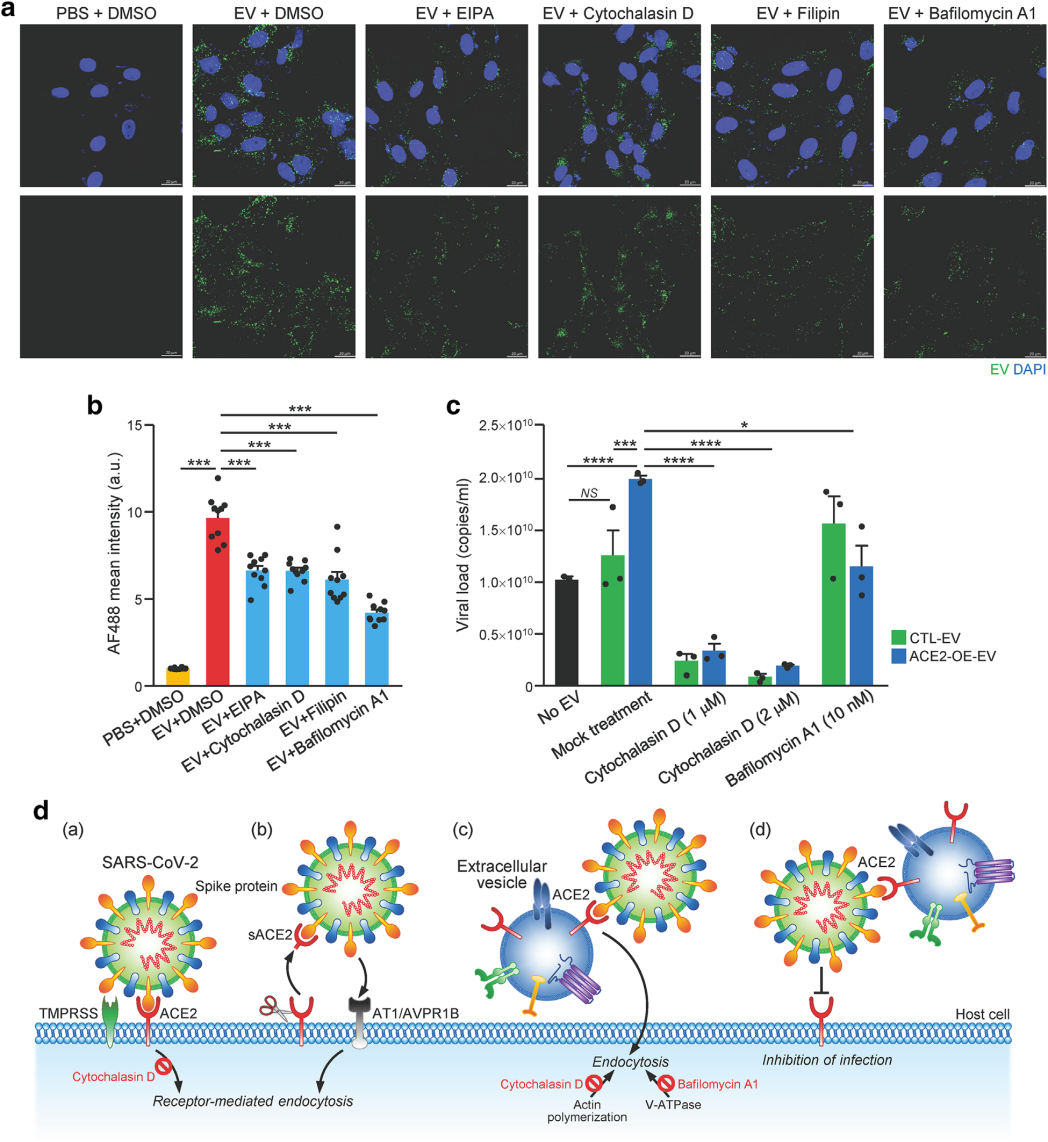
Inhibition of EV uptake reduces infectivity of SARS‐CoV‐2 on Vero E6 cells. (a) Vero E6 cells were pretreated with inhibitors including EIPA, Cytochalasin, Filipin and BafA1 followed by incubation with PKH67‐labeled EVs of 293T ACE2 overexpressing cells. Cells were fixed and stained with DAPI. Representative fluorescent images captured by confocal microscope are shown. Scale bar: 20 μm. (b) The fluorescent signal was analysed using ZEN Software Version 3.1. Error bars represent mean ± SEM. ***P < 0.001. (c) Vero E6 cells were infected with SARS‐CoV‐2 premixed with the indicated EVs (1:8 dilution) and cytochalasin D and BafA1. The infectious media were collected to determine viral load by RT‐qPCR. Representative data with error bar represents mean ± SEM. Asterisks indicate statistical significance compared with PBS control group. *P < 0.05, ***P < 0.001, ****P < 0.0001. (d) Schematic illustration of the proposed cell entry mechanism of SARS‐CoV‐2 mediated by EV‐ACE2. (a) SARS‐CoV‐2 spike (S) protein binds ACE2 on host cells followed by activation of S protein by host protease TMPRSS2. Cytochalasin D blocks infection of host cells by SARS‐CoA‐2. (b) Proteolytic cleavage of ACE2 releases secretory ACE2 (sACE2) which facilitates virus cell entry through AVPR1B and/or AT1 receptor. (c) ACE2 on the surface of EV binds S protein and assists cell entry of virus through EV uptake mechanism that can be inhibited by cytochalasin D (inhibitor of actin polymerization) and BafA1 (inhibitor of vacuolar H+ ATPase). (d) ACE2 carried by EV acts as a decoy to bind virus, thus limits the cell entry of virus

Cells have been shown to take up EVs by various of endocytic pathways (Mulcahy et al., 2014). To ascertain the infectivity enhancement was promoted by EVs, various inhibitory drugs targeting different routes of EVs uptake were tested on Vero E6 cells with the incubation of PHK67‐labeled ACE2‐OE‐EV (Figure 2a). Specially, EIPA inhibits macropinocytosis, filipin inhibits lipid raft‐mediated endocytosis, Cytochalasin D induces depolymerization of the actin cytoskeleton and Bafilomycin A1 (BafA1) inhibits vacuolar H+ ATPase. Inhibition of EV uptake by Vero E6 cells up to 60% was observed after treating with any of these inhibitors (Figure 2b). As demonstrated in the previous study, BafA1 and cytochalasin D, which impair endosomal acidification and endosomal‐lysosomal system, respectively, inhibit the infection of authentic SARS‐CoV‐2 virus (Yeung et al., 2021). We further tested the effect of these two inhibitors in the infectivity of SARS‐CoV‐2 coupling with ACE2‐OE‐EVs using multicycle virus growth assay. RT‐qPCR analysis of viral copy number showed that the infectivity of virus was significantly enhanced by ACE2‐OE‐EV but not CTL‐EV in mock treatment, indicating that ACE2, expressed on EVs, facilitated infectivity of SARS‐CoV‐2 virus. Reduction of infectivity in CTL‐EV and ACE2‐OE‐EV groups was observed upon cytochalasin D treatment in a dosage dependent manner (Figure 2c). BafA1 treatment also significantly reduced the infectivity of SARS‐CoV‐2 induced by ACE2‐OE‐EV. Taken together, these findings highlight the increased efficiency of ACE2‐enriched EVs to enhance SARS‐CoV‐2 viral entry compared to control EVs with barely detectable ACE2. The enhanced infectivity of SARS‐CoV‐2 induced by ACE2‐OE‐EVs could be reduced by inhibitors that target different entry routes of EVs.

## 4 DISCUSSION

Recently, the contribution of EVs to COVID‐19 is an active field that attracts a lot of attention (Hassanpour et al., 2020; Xia et al., 2021). Antiviral effect of antimalarial drugs has been found to block EVs release, endocytosis and phagolysosomal fusion in cell‐based assay, indicating the involvement of EVs in SARS‐CoV‐19 infection (Kumar et al., 2020; Singh et al., 2021). However, the subsequent clinical trials have failed to show prominent benefits to COVID‐19 patients (Tripathy et al., 2020). Another study reported that ACE2 has been transferred by EVs among various types of cells, implying the possibility of SARS‐CoV‐2 to bind with ACE2 that expressed on EVs (Wang et al., 2020). This has inspired the idea to inhibit EV trafficking as an antiviral approach to SARS‐CoV‐2 infection (Urciuoli & Peruzzi, 2020). Contrarily, our data demonstrate that ACE2‐enriched EVs could enhance the infectivity of SARS‐CoV‐2 in a much more efficient manner than viruses alone (Figure 2d). Thus, ACE2‐enriched EVs represent a ‘trojan horse’ for SARS‐CoV‐2 to enter host cells. Subsequent blockage of EVs uptake by different inhibitors including cytochalasin D and BafA1, could reduce the infectivity of SARS‐CoV‐2. This implicates the binding complex of SARS‐CoV‐2 virus and EVs possibly enters host cells through different routes, in a much more complicated way than what we have proposed (Figure 2d).

The contribution of cytoskeleton remodelling to SARS‐CoV‐2 replication has been shown by treating cells with Withaferin A, a compound that disrupts the intermediate filaments network, in which robust reduction in viral replication and amount of infectious virus released from cells has been observed (Cortese et al., 2020). In same study, treatment with another agent that disrupt microfilament, lantrunculin A, does not alter viral replication or live virus production, highlighting the limited role of the actin network to SARS‐CoV‐2 replication. Another study has shown that BafA1 exhibits greatest potential to inhibit SARS‐CoV‐2 infection compared to cytoskeletal drugs, jasplakinolide and cytochalasin D (Yeung et al., 2021). These observations suggest that endosomal acidification plays a significant role in receptor‐mediated cell entry of SARS‐CoV‐2 and the study has further shown the involvement of dynamin 2 (Dyn2)‐dependent endocytosis pathway in facilitating entry of SARS‐CoV‐2 into host cells. Indeed, the importance of endosomal acidification for SARS‐CoV‐2 has been corroborated by using an antiviral peptide to inhibit endosomal acidification (Zhao et al., 2020) and BafA1 to inhibit SARS‐CoV‐2 cell entry (Zhang, Wang et al., 2021). On the contrary, BafA1 neutralizes the acidification of endosomes and lysosomes leading to the inhibition of EV degradation and enhancement of exosome release (Cashikar & Hanson, 2019; Edgar et al., 2016; Mathieu et al., 2021). Our findings showed that BafA1 inhibited the infectivity of SARS‐CoV‐2 induced by ACE2‐OE‐EV when compared to nontreated cells, suggesting BafA1 abolishes the inducing effect of ACE2‐OE‐EV. Such reduction was not observed in CTL‐EV group after BafA1 treatment, indicating BafA1 does not affect the virus infectivity per se. However, the lack of understanding about the mechanism underlying the entry route utilized by SARS‐CoV‐2 mediated by EVs limits the interpretation of the effect of BafA1 on endocytosis. When cytochalasin D was used to treat cells, a marked reduction of infectivity in both CTL‐EVs and ACE2‐OE‐EVs group was observed. This cytoskeletal inhibitor has been shown to inhibit both SARS‐CoV‐2 viral infection and EVs uptake (Mulcahy et al., 2014; Zhang, Wang et al., 2021). CTL‐EV did not enhance viral infection as shown in Figure 2c; thus, the reduction of infection of CTL‐EV‐treated cells by cytochalasin D could be due to inhibiting the entry of virus into cells. This implies that the overall compromised infectivity of virus on ACE‐OE‐EV‐treated cells by cytochalasin treatment could be resulted from blockade of cell entry of virus alone or EV‐bound virus. Further studies to determine the detailed molecular mechanisms underlying the entry route utilized by SARS‐CoV‐2 and EVs need to be conducted.

Intriguingly, ACE2‐expressing EVs have also inspired a competitive inhibition therapy against SARS‐CoV‐2, which proposes to use these EVs to occupy SARS‐CoV‐2 S protein S1 domain, with the aim to protect cells from viral infection (Inal, 2020). As demonstrated in several studies that mainly used RBD binding assay and pseudoviruses infection, authentic or engineered ACE2‐expressing EVs have been shown to exhibit a potential therapeutic effect to block SARS‐CoV‐2 infection (Cocozza et al., 2020; Rao et al., 2020; Wu et al., 2022; Xie et al., 2021; Zhang, Jeppesen et al., 2021; Zhang, Huang et al., 2021). It is worth noting that engineered cell‐mimicking nanodecoys with ACE2 also employed similar strategy to neutralize SARS‐CoV‐2 infection (Li et al., 2021; Rao et al., 2020). Nonetheless, EVs tagged with receptor‐binding domain (RBD) of the viral spike protein have been demonstrated to bind to ACE2‐expressing cells specifically and efficiently to deliver antiviral agents loaded within EVs against SARS‐CoV‐2 infection (Fu & Xiong, 2021). However, another study showed that SARS‐CoV‐2 spike protein carrying EVs function as decoy targets for convalescent patient serum‐derived neutralizing antibodies and reduces their effectiveness in blocking viral entry (Troyer et al., 2021).

As highlighted by the International Society for EV (ISEV) and for Cellular and Gene Therapies (ISCT) recently, EV‐based therapeutic development for COVID‐19 will have to meet several important criteria and safety regulations before implementing in human subjects (Borger et al., 2020). Our data revealed a distinct role of EVs in enhancing the infectivity of SARS‐CoV‐2, which is opposing to the strategy to employ EVs, especially ACE2‐EV, as a therapeutic option for SARS‐CoV‐2 infection. The lack of clarity in experimental details in previous studies, especially the concentration of extracellular vesicles used, has been a stumbling block for us to understand and make fairer comparison between different sets of data and ours. In comparison to a study which provides working concentration of EVs, 1 × 10^9^ ‐ 1 × 10^10^ particles/ml were used versus 3.13 × 10^8^ ‐ 5 × 10^9^ particles/ml used in current study (Cocozza et al., 2020). The disparity might also be derived from the use of different cell lines, EVs collection method and infection method. The use of 293FT‐ACE2, Caco‐2 and Calu‐3 cell lines for infection is also different from commonly used Vero E6 cell line in our study (Cocozza et al., 2020). Indeed, Caco‐2 and Calu‐3 have been shown previously to have a very low susceptibility to SARS‐CoV‐2 infection compared to Vero E6 cells (Yeung et al., 2021). It will be of great interest to investigate further the role of ACE2‐EVs in SARS‐CoV‐2 infection, especially in a sense of physiological relevance, by employing different human cell lines and animal models. Another important criterion to be considered is the use of pseudovirus in previous studies compared to authentic virus in our study. Pseudoviruses are normally made by transfection of lentiviral backboned plasmid with expression of SARS‐CoV‐2 spike protein, thus serves as an important surrogate and harmless platform for SARS‐CoV‐2 study. The disadvantage of pseudovirus is that it only focuses on the properties related to the spike protein but not beyond the processes that spike is responsible for. The use of live virus herein, which is able to propagate and release new copies of virus, coupling with ACE2‐EVs in the environment, might form a positive loop to further enhance the subsequent rounds of infection and suggest an unexplored role of ACE2‐EVs in SARS‐CoV‐2 infection cycle. Therefore, we strongly suggest further in‐depth studies to be performed to fully understand the multifaceted role of EVs in COVID‐19, before making EVs as a therapeutic option for COVID‐19. Also, the detailed declaration of experimental design of these EVs‐related SARS‐CoV‐2 studies is much needed and advocated, to allow direct and fairer comparison as well as the reproducibility of experiments.

Besides facilitating the entry of SARS‐CoV‐2 into host cells, EVs may also act as a key mediator to spread SARS‐CoV‐2 particles or components directly. Previous studies have reported the circulating EVs isolated from patients infected with respiratory viruses including rhinovirus and respiratory syncytial virus contained viral antigens (Gunasekaran et al., 2020). Another study also showed that EVs released by SARS‐CoV‐2 genes expressing lung epithelial cells contain viral RNA (Kwon et al., 2020). Besides that, S protein has been loaded into EVs by ectopic expression in 293T cells, which in turn facilitates the interaction of SARS‐CoV‐2 with target cells (Kuate et al., 2007). It is hardly surprising that SARS‐CoV‐2 infected cells could produce EVs containing virus components to boost the spreading of virus. These EVs could function as vehicles for the en bloc delivery of viral particles to further enhance the infection in the microenvironment. Perhaps, ACE2‐OE‐EVs facilitate the infection of SARS‐CoV‐2 and infected cells released both new viruses and EVs that contain viral components, to further augment the subsequent infection. Although this hypothesis is not tested in current study, such possibility would warrant further investigation to understand the complete viral infection cycle that involves EVs.

## CONFLICT OF INTEREST

The authors report no conflict of interest.

## AUTHOR CONTRIBUTIONS

Conceptualization: Sze Keong Tey, Judy Wai Ping Yam; Investigation: Sze Keong Tey, Hoiyan Lam, Samuel Wan Ki Wong, Hanjun Zhao; Writing: Sze Keong Tey, Kelvin Kai‐Wang To, Judy Wai Ping Yam; Funding acquisition: Kelvin Kai‐Wang To, Judy Wai Ping Yam.

## FUNDING INFORMATION

Consultancy Service for Enhancing Laboratory Surveillance of Emerging Infectious Diseases and Research Capability on Antimicrobial Resistance for Department of Health of the HKSAR and The University of Hong Kong Seed Funding for Strategic Interdisciplinary Research Scheme, 102009863 and 007000142.

## Supporting information

Supporting Information
