# Supplementary material for: ACE2‐enriched extracellular vesicles enhance infectivity of live SARS‐CoV‐2 virus
Source: J Extracell Vesicles. 2022 May 18;11(5):e12231. doi: 10.1002/jev2.12231 (PMC9115585; doi:10.1002/jev2.12231)
Supplement: Supplementary file 1 — Supporting Information [file JEV2-11-0-s001.docx]

**ACE2-enriched extracellular vesicles enhance infectivity of live SARS-CoV-2 virus**

Sze Keong Tey^1, 2^, Hoiyan Lam^3^, Samuel Wan Ki Wong^1^, Hanjun Zhao^3^, Kelvin Kai-Wang To^2,*^, Judy Wai Ping Yam^1,*^

^1^Department of Pathology, School of Clinical Medicine, Li Ka Shing Faculty of Medicine, The University of Hong Kong, Pokfulam, Hong Kong Special Administrative Region, People’s Republic of China

^2^School of Biological Sciences, College of Science, Nanyang Technological University, Singapore 637551, Singapore

^3^State Key Laboratory for Emerging Infectious Diseases, Carol Yu Centre for Infection, Department of Microbiology, School of Clinical Medicine, Li Ka Shing Faculty of Medicine, The University of Hong Kong, Pokfulam, Hong Kong Special Administrative Region, People’s Republic of China

Supplementary Information


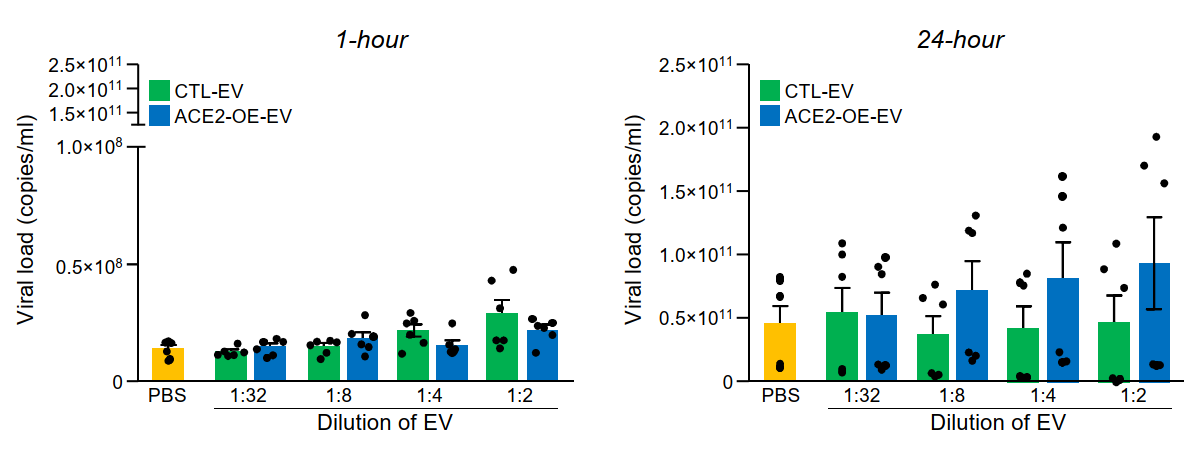
**Supplementary Figure S1.** ACE2-enriched EVs increase infectivity of live SARS-CoV-2 on Vero E6 cells. (A) Vero E6 cells were infected with SARS-CoV-2 pre-mixed with the indicated diluted EVs. The infectious media were collected to determine viral load by RT-qPCR. The data represent the mean of 2 independent experiments of absolute viral load with 3 biological samples each. Error bars represent mean ± SEM. Asterisks indicate statistical signiﬁcance compared with PBS control group. ***P* < 0.01, *****P* < 0.0001. *NS*, Not significant.
